# Early Childhood Developmental Status in Low- and Middle-Income Countries: National, Regional, and Global Prevalence Estimates Using Predictive Modeling

**DOI:** 10.1371/journal.pmed.1002034

**Published:** 2016-06-07

**Authors:** Dana Charles McCoy, Evan D. Peet, Majid Ezzati, Goodarz Danaei, Maureen M. Black, Christopher R. Sudfeld, Wafaie Fawzi, Günther Fink

**Affiliations:** 1 Department of Global Health and Population, T. H. Chan School of Public Health, Harvard University, Boston, Massachusetts, United States of America; 2 Graduate School of Education, Harvard University, Cambridge, Massachusetts, United States of America; 3 MRC-PHE Centre for Environment and Health, School of Public Health, Imperial College London, London, United Kingdom; 4 Department of Epidemiology, T. H. Chan School of Public Health, Harvard University, Boston, Massachusetts, United States of America; 5 School of Medicine, University of Maryland, Baltimore, Baltimore, Maryland, United States of America; 6 RTI International, Research Triangle Park, North Carolina, United States of America; Makerere University Medical School, UGANDA

## Abstract

**Background:**

The development of cognitive and socioemotional skills early in life influences later health and well-being. Existing estimates of unmet developmental potential in low- and middle-income countries (LMICs) are based on either measures of physical growth or proxy measures such as poverty. In this paper we aim to directly estimate the number of children in LMICs who would be reported by their caregivers to show low cognitive and/or socioemotional development.

**Methods and Findings:**

The present paper uses Early Childhood Development Index (ECDI) data collected between 2005 and 2015 from 99,222 3- and 4-y-old children living in 35 LMICs as part of the Multiple Indicator Cluster Survey (MICS) and Demographic and Health Surveys (DHS) programs. First, we estimate the prevalence of low cognitive and/or socioemotional ECDI scores within our MICS/DHS sample. Next, we test a series of ordinary least squares regression models predicting low ECDI scores across our MICS/DHS sample countries based on country-level data from the Human Development Index (HDI) and the Nutrition Impact Model Study. We use cross-validation to select the model with the best predictive validity. We then apply this model to all LMICs to generate country-level estimates of the prevalence of low ECDI scores globally, as well as confidence intervals around these estimates.

In the pooled MICS and DHS sample, 14.6% of children had low ECDI scores in the cognitive domain, 26.2% had low socioemotional scores, and 36.8% performed poorly in either or both domains. Country-level prevalence of low cognitive and/or socioemotional scores on the ECDI was best represented by a model using the HDI as a predictor. Applying this model to all LMICs, we estimate that 80.8 million children ages 3 and 4 y (95% CI 48.1 million, 113.6 million) in LMICs experienced low cognitive and/or socioemotional development in 2010, with the largest number of affected children in sub-Saharan Africa (29.4.1 million; 43.8% of children ages 3 and 4 y), followed by South Asia (27.7 million; 37.7%) and the East Asia and Pacific region (15.1 million; 25.9%). Positive associations were found between low development scores and stunting, poverty, male sex, rural residence, and lack of cognitive stimulation. Additional research using more detailed developmental assessments across a larger number of LMICs is needed to address the limitations of the present study.

**Conclusions:**

The number of children globally failing to reach their developmental potential remains large. Additional research is needed to identify the specific causes of poor developmental outcomes in diverse settings, as well as potential context-specific interventions that might promote children’s early cognitive and socioemotional well-being.

## Abstract 2

Using survey data from 35 low- and middle-income countries, Dana McCoy and colleagues estimate the prevalence of children who are reported by their caregivers to show low cognitive and/or socioemotional development.

## Author Summary

**Why Was This Study Done?:**

- Previous research suggests that more than 200 million children under age five living in low- and middle-income countries (LMICs) experience malnutrition and poverty.
- Despite this known risk, little is known about the status of young children’s cognitive and socioemotional development.
- Estimates of the number of children facing developmental setbacks are important for policy and resource allocation, as well as for tracking progress toward meeting global development goals (for example, the Sustainable Development Goals).

**What Did the Researchers Do and Find?:**

- This study extrapolates data from nearly 100,000 three- and four-year-old children living in 35 LMICs sampled as part of the Multiple Indicator Cluster Survey and the Demographic Health Surveys.
- The results suggest that one in every three preschool-aged children living in LMICs is failing to meet basic milestones in either their cognitive or socioemotional development, with an additional 16% facing setbacks in their physical growth.

**What Do These Findings Mean?:**

- The results highlight the ongoing challenges faced by young children living in developing countries in meeting basic developmental milestones.
- Additional intervention is needed to improve children’s cognitive and socioemotional well-being worldwide.
- Future research should consider additional dimensions of children’s development across a broader age range.

## Introduction

The early years of life are critical for children’s development of foundational cognitive and socioemotional characteristics. Between birth and age 5 y, children develop a set of age-appropriate core cognitive skills that allow them to maintain attention, understand and follow directions, communicate with others, and solve progressively more complex problems. Children’s experiences of early warm and responsive relationships with caregivers and peers can also help them to develop foundational social and emotional competencies, including the ability to get along with others and independently manage negative emotions and aggressive behaviors [1–3]. These early patterns are essential for achieving subsequent developmental milestones [4–6], for ensuring both mental and physical health, and, ultimately, for becoming economically successful and productive adults [3,7,8].

In a seminal paper opening the 2007 *The Lancet* series on early child development (ECD), Grantham-McGregor et al. [2] combined country-level data on stunting and poverty from UNICEF and the World Bank to estimate that 219 million children under the age of 5 y in 2004 were not reaching their developmental potential. Although poverty and stunting are critical predictors of children’s short- and long-run well-being [9,10], they explain to only a limited degree the variance in children’s early cognitive and socioemotional characteristics. While major progress has been made over the past several decades to measure and reduce the prevalence of physical growth deficits and poverty in low- and middle-income countries (LMICs), population-level data on cognitive and socioemotional development have, until recently, remained limited due to the conceptual, technical, and cultural challenges of collecting data on complex developmental processes across diverse yet low-resourced settings. At the same time, emerging evidence from high-income countries suggests that population-level measures can be useful not only for quantifying ECD but also for predicting later-life academic, social, and emotional well-being [11,12].

The aim of the present paper is to estimate the number of preschool-aged children in LMICs with low cognitive and/or socioemotional development using newly available population-representative data collected as part of UNICEF’s Multiple Indicator Cluster Survey (MICS) program and the Demographic and Health Surveys (DHS) program. For the purposes of this paper, we define low cognitive development as an inability to follow simple directions and work independently, and low socioemotional development as an inability to control aggression, avoid distraction, and/or get along well with other children. We base these definitions on available data from the Early Childhood Development Index (ECDI), the first widely available tool for measuring the early development of 3- and 4-y-old children at the population level. Although necessarily limited in the breadth and depth of its content, the ECDI’s global coverage and inclusion of developmental characteristics that are particularly amenable to early intervention provide an important opportunity for informing global ECD policy. In the present paper, we leverage this opportunity by combining ECDI data from 35 nationally representative datasets to estimate the prevalence of low cognitive and socioemotional development scores in each country, as well as to model the relationship between developmental status in both domains and other country-level characteristics. Based on this modeled relationship, we are then able to provide national and regional estimates of the number of children with low cognitive and socioemotional ECDI scores in LMICs.

## Methods

### Data

For the proposes of this study, we combined all available ECDI data from the DHS and MICS programs. Both MICS and DHS surveys follow a two-stage cluster random sampling procedure, randomly selecting households with children under the age of 5 y in a representative set of enumeration areas typically drawn from a national census. The 35 surveys used in the present analysis were chosen from the larger MICS and DHS programs based on the following criteria: (1) the surveyed country was classified as a low- or middle-income country in 2010, (2) both child anthropometric and early development measures were available, (3) a nationally representative (rather than subnational) sampling frame was used, and (4) data were publicly available prior to February 5, 2016. Descriptive data on these 35 countries can be found in S1 Table.

#### Ethical considerations

This study was deemed exempt from ethics review by the Harvard School of Public Health Institutional Review Board, as no human participants work was conducted as part of this project. All data used are in the public domain and fully de-identified.

#### Measures of cognitive and socioemotional development

The primary source of child development data was the ECDI, administered as part of the fourth and fifth rounds of the MICS and as part of wave VI of the DHS. The ECDI is a caregiver-reported index of ten yes/no questions designed for children ages 36 to 59 mo to assess four domains of development: literacy-numeracy, learning/cognition, physical development, and socioemotional development. These ten survey items were selected from an original list of 158 items generated by child development experts following a multistage, multicountry pilot and validation process. Items were chosen for inclusion in the final version of the ECDI based on their test-retest and inter-rater reliability and their validity against existing, previously validated measurement tools, and were grouped into domains based on factor analysis [13,14]. Confirmatory factor analysis within the current sample indicated adequate model fit of the original domains proposed by the ECDI developers: χ^2^(29) = 3993.01, *p* < 0.001; root mean square error of approximation = 0.04; CFI = 0.99; standardized root mean square residual = 0.02 [15]. Factor loadings of each item within each domain are shown in S2 Table. When replicated within each country, the results of the confirmatory factor analysis—including both fit and factor loadings—were relatively stable.

For the purpose of this paper, we focused on items from the cognitive and socioemotional domains of the ECDI only. The characteristics captured in these domains—including comprehension of directions and ability to work independently in the cognitive domain, and ability to control aggressive behaviors, avoid distraction, and get along with peers in the socioemotional domain—are core milestones of early childhood that are strongly related to later life outcomes [2,3,8,16,17]. Deficits in these areas are commonly considered as signs of developmental problems in the pediatric literature [18,19]. The two other domains covered in the ECDI questionnaire and shown in Table 1 (physical and literacy-numeracy) were excluded from this paper for several reasons. Although the three items on literacy and numeracy are relevant indicators of pre-academic knowledge, observed differences in this domain are more likely to reflect differences in countries’ social/cultural norms around early education than they are likely to reflect children’s cognitive capacity. Furthermore, the ECDI literacy-numeracy items are substantially more advanced than the types of pre-academic skills captured in developmentally comparable tools (e.g., the Ages & Stages Questionnaire, the Malawi Developmental Assessment Tool), which focus, for example, on basic counting but not on recognition of numeric symbols. The opposite holds true for the pincer grasp in the physical domain, which represents a skill typically acquired before 12 mo of age and which therefore would capture only very severe developmental setbacks in the 3- to 4-y age range. The second item in the physical domain—being “too sick to play”—was excluded because it represents children’s health status rather than their early developmental skills.

**Table 1:** Early Childhood Development Index items.

Domain and Item	Included in Present Study?	Construct Measured	Age Appropriate?	Similar Item Included in ASQ-III or SDQ for 36-to 60-mo Age Range?	Failure of Attainment Acknowledged by AAP as “Possible Sign of Developmental Delay” for Ages 3–4 y?	Rationale for Inclusion/Exclusion
**Literacy-numeracy**						Measures of academic knowledge rather than general capacity; items too difficult for young children
Can your child identify or name at least ten letters of the alphabet?	No	Early literacy	No	ASQ-III (60 mo), though four letters only	No	
Can your child read at least four simple, popular words?	No	Early literacy	No	No	No	
Does your child know the name and recognize the symbol of all numbers from 1 to 10?	No	Early numeracy	No	ASQ-III (54–60 mo), though two numbers only	No	
**Learning/cognition**						Age-appropriate measures of cognition
Does your child follow simple directions on how to do something correctly?	Yes	Cognition	Yes	ASQ-III (36–60 mo)	Yes, “cannot understand two-part commands”	
When given something to do, is your child able to do it independently?	Yes	Cognition	Yes	No	No, though “shows more independence” listed as positive milestone	
**Physical development**						“Too sick to play” not a measure of development; pincer grasp appropriate for under 12 mo
Is your child sometimes too sick to play? (reverse coded)	No	Health	Yes	No	No	
Can your child pick up a small object with two fingers, like a stick or a rock from the ground?	No	Fine motor	No	No	Yes, “cannot grasp a crayon between thumb and fingers”	
**Socioemotional development**						Age-appropriate measures of socioemotional development
Does your child kick, bite, or hit other children or adults? (reverse coded)	Yes	Aggressive behavior	Yes	SDQ (36–60 mo), focus on fighting/bullying	Yes, “exhibits aggressive behavior”	
Does your child get easily distracted? (reverse coded)	Yes	Attention	Yes	SDQ (36–60 mo)	Yes, “is easily distracted”	
Does your child get along well with other children?	Yes	Social competence	Yes	ASQ-III (60 mo), focus on sharing and taking turns; SDQ (36–60 mo), focus on being well liked	Yes, “shows little interest in playing with other children”	

All ECDI items scored as yes (one)/no (zero). Children were classified as “low development” for a domain if they received a score of zero on more than one item within the domain.

The Ages & Stages Questionnaire, Third Edition, and the Strengths and Difficulties Questionnaire were chosen as comparators because they are well-validated measures of early development used across high-, middle-, and low-income country contexts.

AAP, American Academy of Pediatrics; ASQ-III, Ages & Stages Questionnaire, Third Edition; SDQ, Strengths and Difficulties Questionnaire.

All five items in the cognitive and socioemotional domains of the ECDI were found to match conceptually with items from existing, validated early childhood assessments. Each of them was also considered to be developmentally appropriate across the 3- to 4-y age range, in the sense that they reflect general skills and behaviors that are important within this relatively wide age period rather than specific developmental milestones that would be suitable for a more limited age range. Within each domain, we followed the guidelines developed for the original ECDI questionnaire by considering a child to have a low ECDI score in a domain if the child failed (i.e., scored zero on) more than one item in the domain. Observations with missing ECDI data were excluded from our analysis.

#### Other data

In addition to the ECDI, several other characteristics from the MICS and DHS were used for the analysis, including children’s sex, stunting status (based on a height-for-age of <2 standard deviations below the WHO standard), household wealth quintile, urbanicity, age, and cognitive stimulation quintile (based on a sum of six caregiver-reported items regarding whether an adult in the household read to, played with, told stories to, counted with, sang to, or traveled outside of the home with the child). In addition to the MICS and DHS, we used 2010 data on countries’ prevalence of stunting from the Nutrition Impact Model Study [20] and data on life expectancy, education, and income from the Human Development Index (HDI) project [21].

### Statistical Analysis

We estimated the prevalence of low cognitive and/or socioemotional ECDI scores separately for each of the 35 sample countries. We also estimated the prevalence of low development scores by sex, stunting status, household wealth quintile, urbanicity, age (3 versus 4 y), and cognitive stimulation quintile. We produced these prevalence estimates using sampling weights provided by the MICS and DHS that account for clustering, selection, and stratification in order to ensure that estimates are nationally representative.

In order to generate global estimates of the number of children with low development according to the ECDI, we developed country-level prediction models based on ordinary least squares regression. The coefficients of these models were estimated using data from countries with MICS or DHS surveys in which both ECDI scores and the levels of predictors were known. Cross-validation was used to select the best-fitting model. In the cross-validation process, parts of the available sample are intentionally excluded from the original model fitting; the predicted values of the model are then compared to the actually observed values in the excluded sample to assess average predictive errors. Following the algorithms described in Arlot and Celisse [22], we implemented data splitting for training set sizes *n −* 1 and *n −* 2. Squared prediction errors were used as the criterion to select the minimum contrast estimator.

We then used the selected model to predict the percentage of children with low cognitive and/or socioemotional development in all LMICs. To obtain global estimates of the absolute number of preschool-aged children with low cognitive and/or socioemotional development according to the ECDI, predicted prevalence rates of low development scores were multiplied by the number of 3- and 4-y-old children in each country in 2010 using data from *World Population Prospects* [23]. Cross-validation errors were used to create 95% confidence intervals around our projections.

To examine whether the 35 sample countries were different from other LMICs, we compared the HDI scores, life expectancy, mean number of years of schooling, and gross national income per capita between the included and excluded countries for 2014. All analyses were conducted using Stata 14 software.

## Results

**Fig 1:**
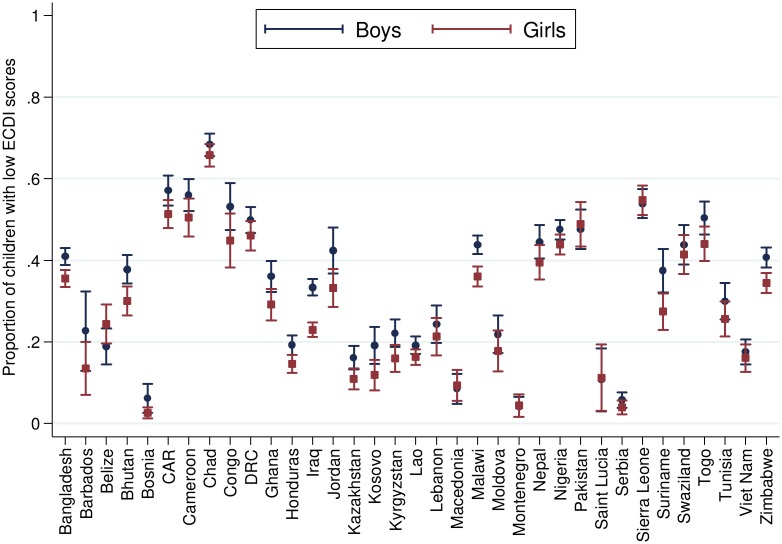
Percentage of children scoring low in cognitive and/or socioemotional development on the ECDI by sex (*r* = −0.04, *p* < 0.01). Correlation performed with girls = 1, boys = 0. CAR, Central African Republic; DRC, Democratic Republic of the Congo; Lao, Lao People’s Democratic Republic; Moldova, Republic of Moldova.

**Fig 2:**
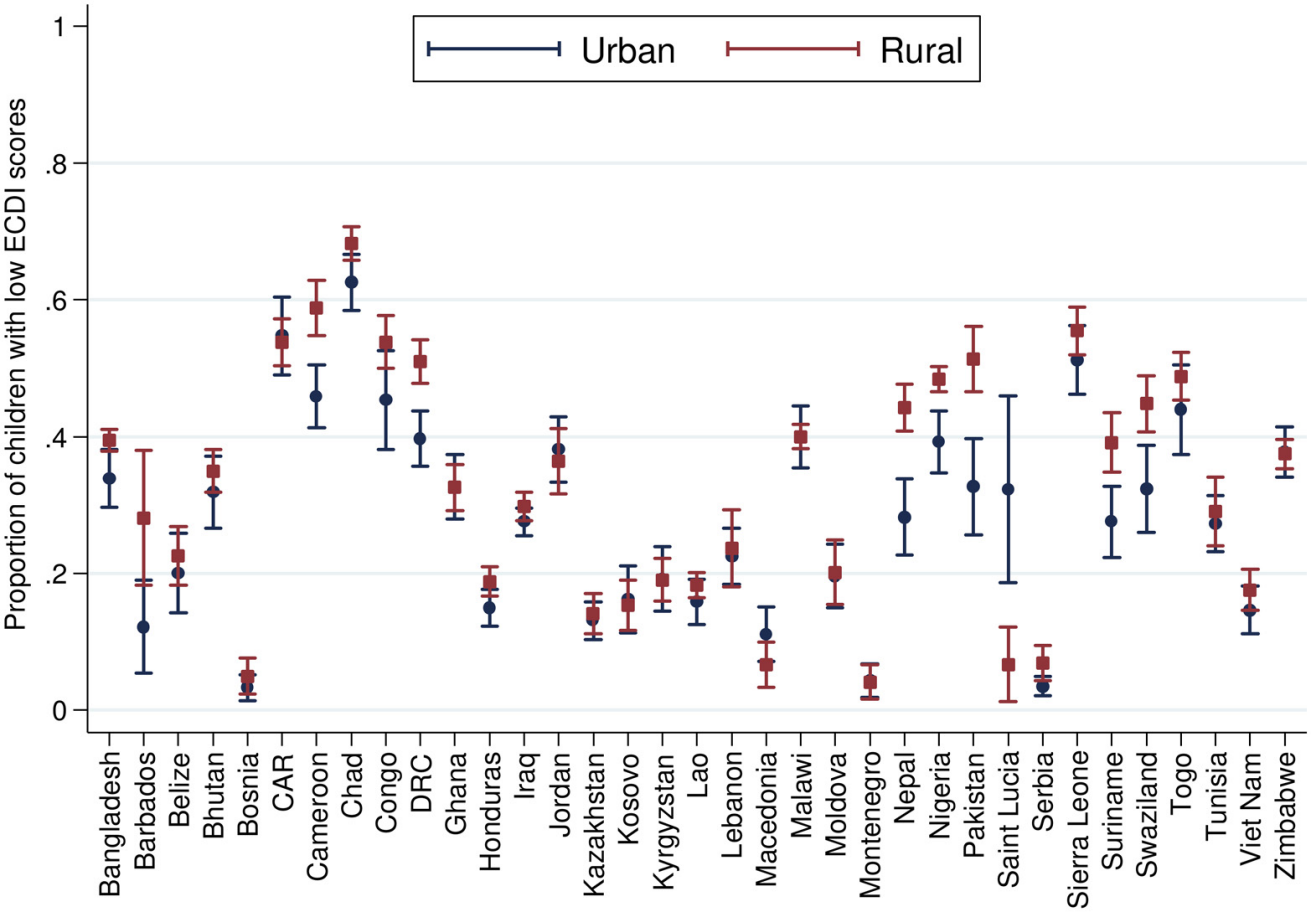
Percentage of children scoring low in cognitive and/or socioemotional development on the ECDI by urbanicity (*r* = 0.07, *p* < 0.01). Correlation performed with rural = 1, urban = 0.

**Fig 3:**
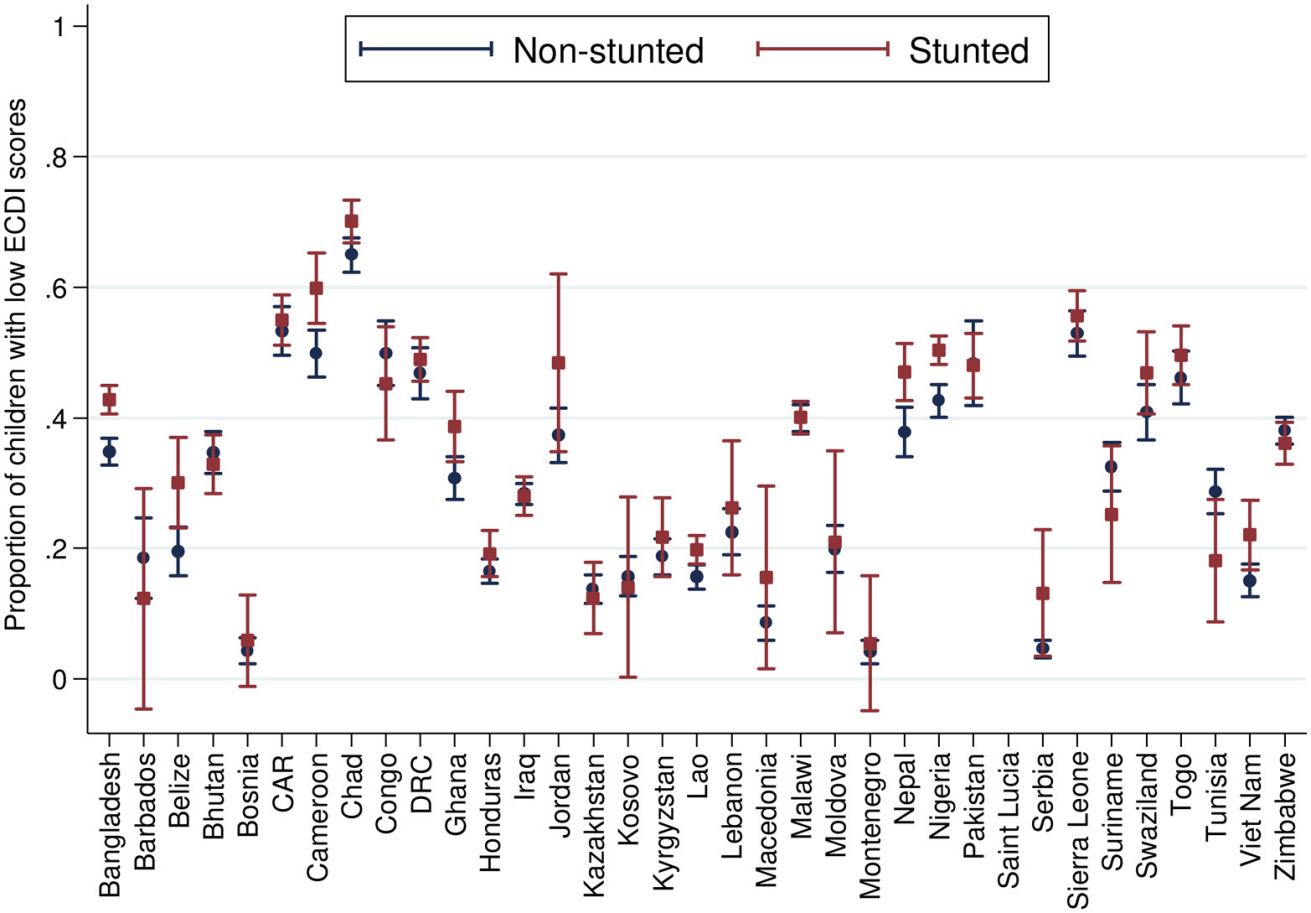
Percentage of children scoring low in cognitive and/or socioemotional development on the ECDI by stunting status (*r* = 0.10, *p* < 0.01). Correlation performed with stunted children = 1, non-stunted children = 0.

**Fig 4:**
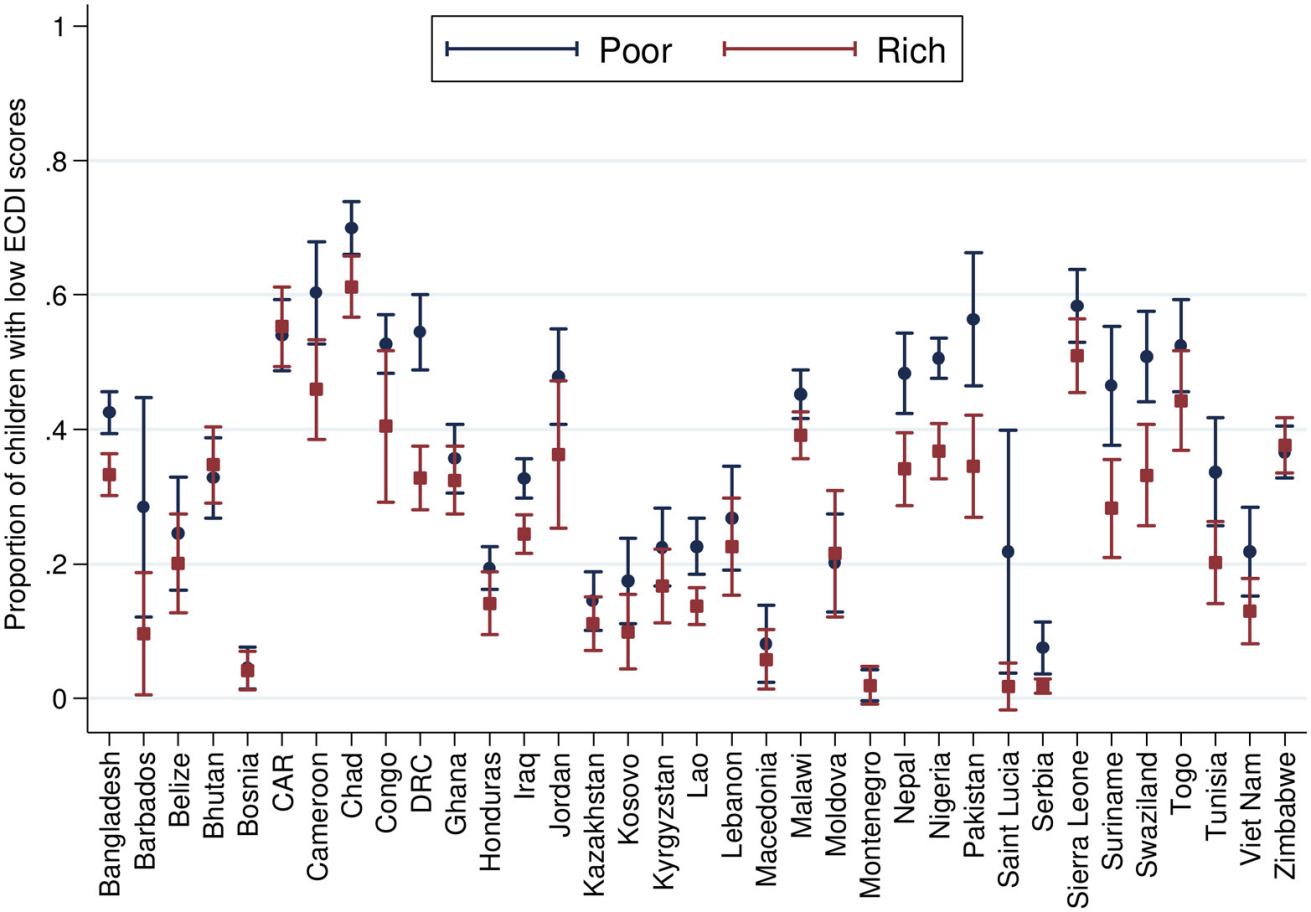
Percentage of children scoring low in cognitive and/or socioemotional development on the ECDI by wealth quintile (*r* = −0.03, *p* < 0.01). Correlation performed with highest wealth quintile = 1, lowest wealth quintile = 0.

**Fig 5:**
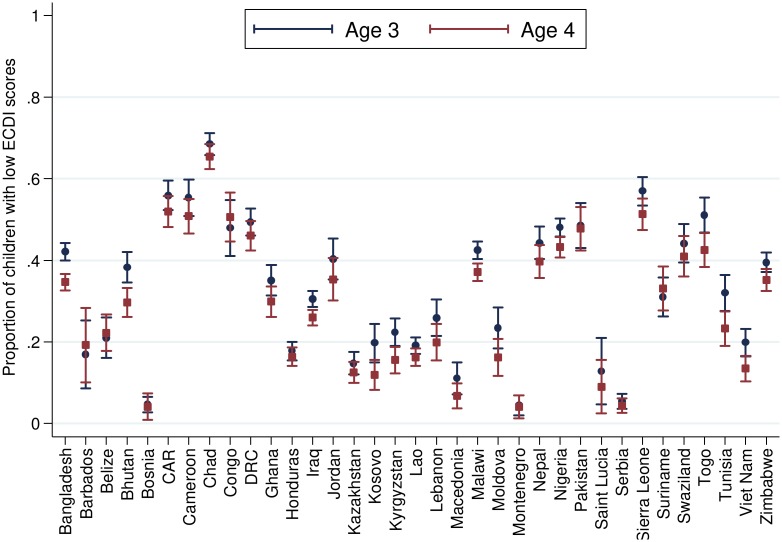
Percentage of children scoring low in cognitive and/or socioemotional development on the ECDI by child age (*r* = −0.05, *p* < 0.01). Correlation performed with children age 4 y = 1, children age 3 y = 0.

**Fig 6:**
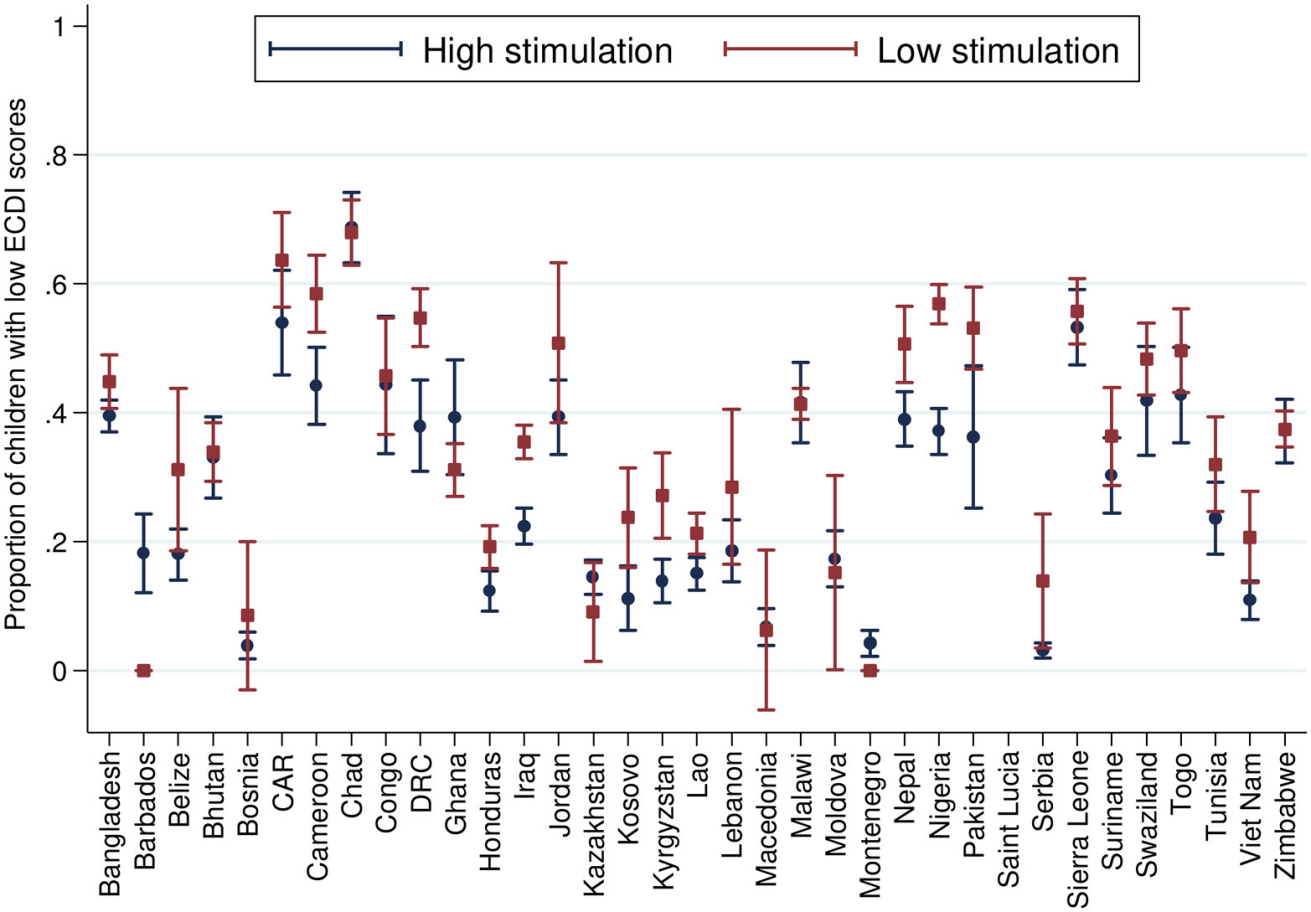
Percentage of children scoring low in cognitive and/or socioemotional development on the ECDI by cognitive stimulation (*r* = 0.06, *p* < 0.01). Correlation performed with lowest quintile of cognitive stimulation = 1, highest quintile of cognitive stimulation = 0.

Table 2 presents the number and percentage of children scoring low in cognitive and/or socioemotional development on the ECDI for each of the 35 sample countries. In the full sample, 35.8% of the 99,222 total children had low cognitive and/or socioemotional ECDI scores, with the highest percentage of low-scoring children in Chad (67.0%), Sierra Leone (54.3%), and Central African Republic (54.1%), and the lowest percentage in Bosnia (4.4%) and Montenegro (4.3%). In most countries it was more common for boys than girls (Fig 1) and children from rural than urban (Fig 2) communities to score low on the ECDI. Consistent with prior work, stunting and low wealth were also associated with low development scores (Figs 3 and 4, respectively). At the same time, the majority of stunted children (55.8%) and the majority of children living in poor households (61.3%) were considered to be developing normally in these domains, whereas approximately one-third of non-stunted children (33.8%) and children from the highest wealth quintile (31.9%) had low ECDI scores. S1 Fig shows children’s height-for-age *z*-scores plotted against their ECDI scores, and demonstrates a strong positive relationship across the height-for-age spectrum. Four-year-olds scored on average slightly better than 3-y-old children, but overall age differences were small (Fig 5). Positive associations were also found between ECDI scores and cognitive stimulation (Fig 6).

**Table 2:** Prevalence of children with low ECDI scores.

Country	Sample Size	Low Cognitive and/or Socioemotional ECDI Score		Low Cognitive ECDI Score		Low Socioemotional ECDI Score	
		*n*	Percent	*n*	Percent	*n*	Percent
Bangladesh	7,713	2,956	38.3%	908	11.8%	2,319	30.1%
Barbados	171	31	18.2%	1	0.4%	30	17.8%
Belize	719	156	21.6%	9	1.2%	150	20.9%
Bhutan	2,200	749	34.1%	145	6.6%	656	29.8%
Bosnia	963	42	4.4%	5	0.6%	37	3.8%
Cameroon	1,587	843	53.1%	247	15.6%	715	45.0%
Central African Republic	3,358	1,817	54.1%	818	24.4%	1,337	39.8%
Chad	4,451	2,982	67.0%	2,347	52.7%	1,308	29.4%
Congo	1,486	729	49.0%	216	14.5%	622	41.9%
Democratic Republic of the Congo	3,726	1,786	47.9%	1,050	28.2%	1,072	28.8%
Ghana	2,928	955	32.6%	309	10.5%	778	26.6%
Honduras	2,800	477	17.0%	34	1.2%	464	16.6%
Iraq	13,119	3,714	28.3%	1,391	10.6%	2,834	21.6%
Jordan	2,597	983	37.8%	257	9.9%	799	30.7%
Kazakhstan	1,686	230	13.6%	80	4.8%	156	9.3%
Kosovo	595	93	15.6%	12	2.1%	85	14.2%
Kyrgyzstan	1,683	321	19.1%	110	6.6%	235	14.0%
Lao People’s Democratic Republic	4,052	719	17.7%	249	6.2%	502	12.4%
Lebanon	695	159	22.9%	48	7.0%	123	17.7%
Macedonia	523	47	8.9%	6	1.2%	42	8.0%
Malawi	7,330	2,930	40.0%	1,338	18.2%	1,986	27.1%
Montenegro	1,206	51	4.3%	9	0.8%	42	3.5%
Nepal	2,142	900	42.0%	378	17.7%	655	30.6%
Nigeria	9,382	4,289	45.7%	1,991	21.2%	3,113	33.2%
Pakistan	1,463	704	48.1%	461	31.5%	381	26.1%
Republic of Moldova	620	124	20.0%	3	0.5%	121	19.5%
Saint Lucia	113	12	11.0%	2	1.5%	12	10.6%
Serbia	3,193	155	4.9%	13	0.4%	146	4.6%
Sierra Leone	3,232	1,755	54.3%	713	22.1%	1,281	39.6%
Suriname	997	319	32.0%	13	1.3%	309	31.0%
Swaziland	1,011	430	42.5%	68	6.8%	388	38.4%
Togo	1,669	789	47.3%	319	19.1%	548	32.8%
Tunisia	1,024	286	27.9%	70	6.9%	249	24.3%
Viet Nam	1,366	229	16.8%	112	8.2%	134	9.8%
Zimbabwe	7,422	2,785	37.5%	759	10.2%	2,338	31.5%
**Total**	99,222	35,547	35.8%	14,492	14.6%	25,967	26.2%

Children were classified as “low development” in an ECDI domain if they received a score of zero on more than one item within the domain.

**Fig 7:**
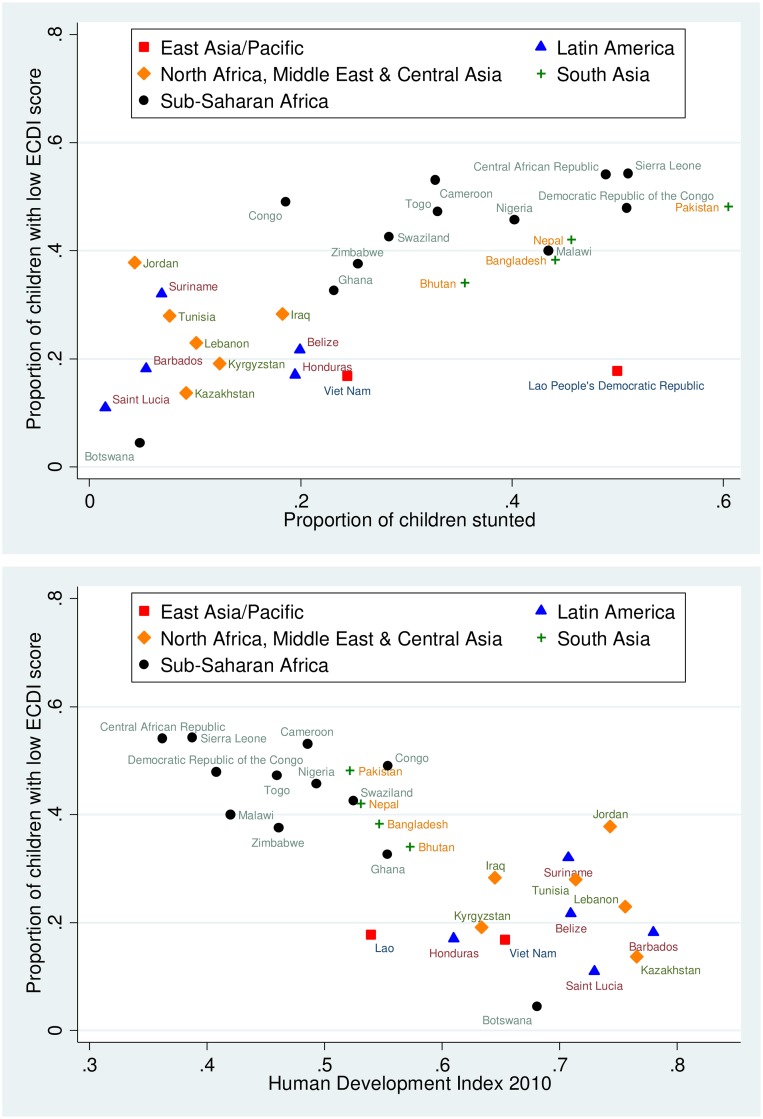
Scatterplots showing country-level relationships between low socioemotional and/or cognitive ECDI score and stunting and HDI. Proportion of children with low socioemotional and/or cognitive ECDI score relative to the proportion of children with stunting (top) and relative to country HDI (bottom).

Fig 7 shows the association between the prevalence of low ECDI scores and stunting, and the association between low ECDI scores and the HDI at the country level. Both correlations are strong, with a correlation coefficient of *r* = 0.72 for the association between the percentage of low-scoring children and the prevalence of stunting, and a correlation coefficient of *r* = −0.84 for the association between the percentage of low-scoring children and the HDI.

In Table 3, we show the results of our country-level multivariate models to predict the percentage of children with low ECDI scores in the 35 sample countries. The model best predicting the country-level prevalence of low ECDI (i.e., the model with the lowest root mean square error both for *n −* 1 and *n* − 2 tests) was the model with HDI as the only predictor (model 2 in Table 3), which we thus selected as our main prediction model.

**Table 3:** Regression models predicting country-level prevalence of low ECDI scores.

Predictors	Percentage of Children with Low Cognitive and/or Socioemotional ECDI Scores		
	Model 1	Model 2	Model 3
Stunting proportion (2010)	0.787*** (0.123)		−0.102 (0.194)
HDI (2010)		−1.063*** (0.112)	−1.159*** (0.202)
Observations	35	35	35
*R* ^2^	0.468	0.700	0.702
Cross validation with *n −* 1 (RMSE)^a^	0.13	0.10	0.10
Cross validation with *n −* 2 (RMSE)^b^	0.09	0.07	0.07

Robust standard errors in parentheses. Model 1 is a linear model that predicts the proportion of children scoring low on the ECDI based on Nutrition Impact Model Study stunting data only. Model 2 is a model using HDI as the only predictor. Model 3 is a model including both predictors. All estimates reflect ordinary least squares estimates with robust standard errors.

^a^Based on all 35 possible permutations of size 34.

^b^Based on 595 permutations of sample size 30.

***p < 0.001.

RMSE, root mean square error.

In S3 Table we compare the countries in the MICS/DHS sample to other LMICs with respect to a range of characteristics and indicators, including education, income, and life expectancy. No statistically significant differences were found at the country-level between the countries in our sample and the 103 LMICs not covered by our data.

In Table 4, we show our global estimates of the number and percentage of children with low cognitive and/or socioemotional development, which suggest that 80.8 million 3- and 4-y-old children (95% CI 48.4 million, 113.6 million) experienced low cognitive and/or socioemotional development in 2010 as measured by the ECDI. This corresponds to a global prevalence in LMICs of 32.9% (95% CI 19.7%, 46.3%). The highest prevalences of low cognitive and/or socioemotional development were estimated for sub-Saharan Africa (43.8%; 95% CI 30.5%, 57.2%) and South Asia (37.7%; 95% CI 24.3%, 51.1%), whereas the lowest prevalences were estimated for the Latin America/Caribbean region (18.7%; 95% CI 5.9%, 32.1%) and the North Africa/Middle East/Central Asia region (18.4%, 95% CI 6.3%, 31.8%). Sub-Saharan Africa and South Asia also account for the majority of predicted children with low development, with an estimated 29.4 (95% CI 20.4, 38.4) and 27.7 (95% CI 17.9, 38.4) million 3- and 4-y-olds, respectively.

**Table 4:** Estimated number of 3- and 4-y-olds with low development according to the ECDI by region.

Region	Total Population ages 3 and 4 y in Millions	Estimated Percentage of Children with Low Cognitive and/or Socioemotional ECDI Scores (95% CI)	Estimated Number of Children with Low Cognitive and/or Socioemotional ECDI Scores in Millions (95% CI)
East Asia/Pacific	58.5	25.9% (12.5%, 39.3%)	15.1 (7.3, 23)
Latin America/Caribbean	21.9	18.7% (5.9%, 32.1%)	4.1 (1.3, 7)
North Africa/Middle East/Central Asia	24.5	18.4% (6.3%, 31.8%)	4.5 (1.5, 7.8)
South Asia	73.4	37.7% (24.3%, 51.1%)	27.7 (17.9, 37.5)
Sub-Saharan Africa	67.0	43.8% (30.5%, 57.2%)	29.4 (20.4, 38.4)
**All LMICs**	245.3	32.9% (19.7%, 46.3%)	80.8 (48.4, 113.6)

Confidence intervals are based on the root mean square errors computed in Table 3. Population numbers are based on the number of children born by country and year in 2010 as reported in *World Population Prospects*: *The 2015 Revision*.

**Fig 8:**
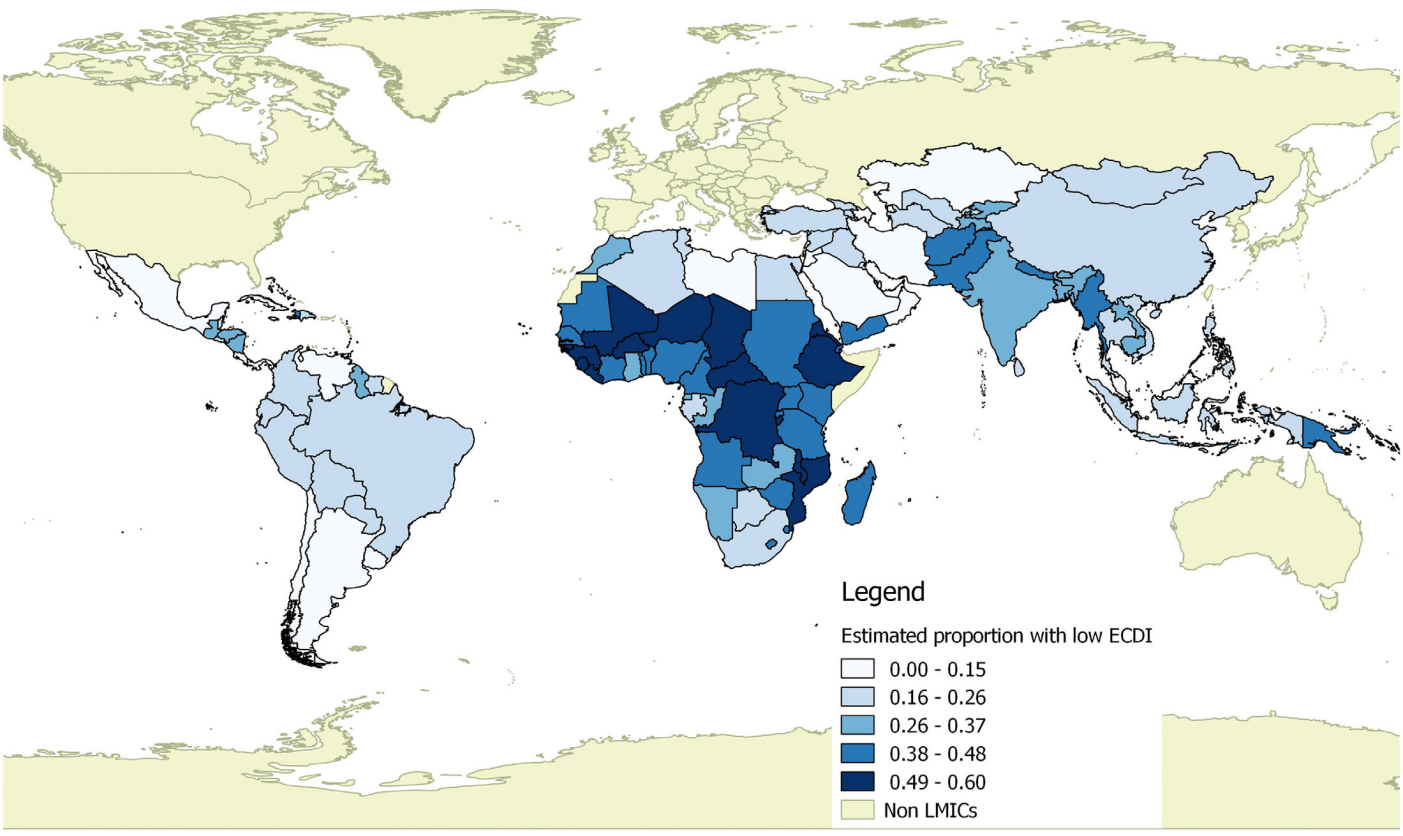
Estimated proportion of children with low development per the ECDI by country. This figure was generated with a shapefile from DIVA-GIS (http://diva-gis.org) using the Open Source Geospatial Foundation’s QGIS package (http://qgis.osgeo.org).

We present the estimated percentage of children with low cognitive and/or socioemotional development by country in S4 Table and Fig 8. The country with the highest estimated number of children with low development was India (17.7 million children), followed by China (6.6 million) and Nigeria (6.0 million). The estimated percentage of children with low cognitive and/or socioemotional development ranged from as few as 4.4% of children in Botswana to 67% of children in Chad.

## Discussion

Our results—which are based on developmentally narrow but nationally representative caregiver reports of children’s early skills and behaviors—suggest that approximately one-third of all 3- and 4-y-old children in LMICs were failing to meet basic cognitive and/or socioemotional milestones in 2010. Low development scores were largely concentrated in areas of the world facing continued high exposure to risk factors such as infectious disease, malnutrition, poverty, and low availability of high-quality healthcare and educational resources. In particular, nearly 38% of children in South Asia and 44% of children in sub-Saharan Africa were estimated to have low development per the ECDI. Additional research is needed to understand the specific sources of developmental issues within these regions; also needed is a potential set of interventions that might be able to mitigate these challenges in the future.

In 2007, Grantham-McGregor et al. [2] estimated that 219 million children—or 39% of the under-5-y population—living in LMICs were failing to meet their developmental potential. The numbers presented in this paper are not directly comparable to these previous estimates, which reflect the total number of children *at risk* of overall developmental disadvantage based on malnutrition and poverty. Our estimates, on the other hand, reflect a more direct quantification of the percentage children with caregiver-reported limitations in cognition and socioemotional functioning, and do not take into account children’s status in other important domains like physical growth. According to the latest global estimates, 29.9% of children under 5 y in LMICs were stunted in 2011 [20]. Our estimates suggest that only 44.2% of stunted children scored low on the ECDI with respect to their cognitive or socioemotional development. Assuming that the rates of stunting in the 3- and 4-y-old population are similar to the rates in the full under-5-y population, an additional 16.7% of 3- and 4-y-olds may be meeting the ECDI’s cognitive and socioemotional milestones yet experiencing physical growth faltering. Combining across estimates, we can therefore estimate that nearly half (49.6%) of 3- and 4-y-old children in LMICs are failing to meet their potential with respect to their cognitive, socioemotional, or physical development.

These results highlight the critical need to consider multipronged approaches to intervention that are able to address the diverse yet relatively common developmental setbacks captured in the ECDI. Research has shown, for example, that the provision of warm, responsive, and stimulating caregiving can effectively promote children’s early cognitive and socioemotional development, even in the presence of risk factors such as poverty and malnutrition [16,24]. Efforts that integrate psychosocial and educational approaches with health and nutrition programming may be particularly promising for promoting ECD, as they are able to target multiple developmental domains while reducing the inefficiencies of independent, multisectoral implementation [25].

We view the use of caregivers’ reports of cognitive and socioemotional development as a major strength of this study, as they allow us to provide global estimates of ECD that are based on specific behaviors and skills, rather than proxy measures such as stunting and poverty. Despite this strength, the work presented also has several important limitations. First, the ECDI items used to quantify children’s development were designed to be brief enough to administer within an existing household survey and general enough to facilitate valid comparison both within the somewhat wide specified age range as well as across diverse linguistic, cultural, and socioeconomic contexts. As a result, the developmental items used in this study are substantially limited in their ability to capture specific subdomains of cognition and socioemotional skills (e.g., pattern recognition, memory, executive function, language skills, emotional competence), culturally based developmental imperatives, and incremental differences in children’s own individual “developmental potential.” Although many of the items included in the ECDI have been used successfully in validated, widely used measures of development (see Table 1), additional work exploring the cross-cultural validity and reliability of these and other developmental milestones is urgently needed, particularly in LMICs [26]. Furthermore, the cutoff for “low” development used in this study has not been sufficiently validated and may not be appropriate for both 3- and 4-y-old children. As a result, the ECDI’s utility as a diagnostic tool is largely unknown. Because of these limitations, the meaning of a low cognitive and/or socioemotional development score must be interpreted in the context of the specific items and thresholds used. Future research is needed to develop additional, more detailed, and age-specific measures of early childhood development that can more accurately capture children’s capacity across a wide range of cultures and local contexts. In addition, work is needed that goes beyond measures of typical development to understand the specific needs of children who may experience more severe disabilities requiring more intensive treatment and care.

A second key strength of our paper is its inclusion of a large and diverse sample of almost 100,000 children from 35 LMICs. At the same time, the MICS and DHS surveys analyzed in this study may not be fully representative of the developing world. The total under-5-y population in the 35 countries analyzed in this paper is approximately 115 million, which corresponds to just over 21% of the current total under-5-y population in LMICs. Data on child development are also available only for children of ages 3 and 4, and it is not clear how similar developmental scores among younger children are to the outcomes observed among 3- and 4-y-olds. Although the average socioeconomic level of countries in the MICS sample is not statistically different from that of countries not covered by the MICS (as shown in S3 Table), additional data spanning the full 0- to 5-y age range are needed to more precisely understand children’s development at the country, regional, and global levels.

Overall, the present study suggests that almost one-third of 3- and 4-y-old children living in LMICs were not meeting basic cognitive and/or socioemotional milestones in 2010, with an additional 16.7% experiencing delayed physical growth (stunting). Programs that aim to reduce poverty, improve nutrition, enhance stable and stimulating caregiving, improve high-quality early educational opportunities, and promote gender equity have the potential to counteract the multitude of risk factors many children continue to experience, and to positively impact children’s developmental outcomes across the life span [16,25,27]. Efforts being led by WHO and UNICEF, for example, have emphasized the importance of integrating broader ECD initiatives within existing community health systems and structures (e.g., the International Developmental Pediatrics Congress). As the international community looks toward a post-2015 Sustainable Development Goal agenda that will inevitably focus on improving young children’s ability to learn and thrive, future efforts must identify additional cost-effective approaches for helping children to achieve their developmental potential, as well as ways to most effectively combine these approaches, ensure their sustainability, and take them to scale.

## Supporting Information

S1 Fig Loess curve with 95% confidence interval showing the relationship between children’s height-for-age *z*-scores and low ECDI scores. (JPG)

S1 Table Sample composition. (DOCX)

S2 Table Factor loadings for ECDI in the full analytic sample. (DOCX)

S3 Table MICS/DHS versus non-MICS/DHS low- and middle-income country characteristics. (DOCX)

S4 Table Estimated percentage and number of children with low development by country. (DOCX)

S1 Text STROBE checklist. (DOC)

## Abbreviations

DHS: Demographic and Health Surveys
ECD: early child development
ECDI: Early Childhood Development Index
HDI: Human Development Index
LMICs: low- and middle-income countries
MICS: Multiple Indicator Cluster Survey
