# Correction: Early Childhood Developmental Status in Low- and Middle-Income Countries: National, Regional, and Global Prevalence Estimates Using Predictive Modelling

**DOI:** 10.1371/journal.pmed.1002233

**Published:** 2017-01-30

**Authors:** Dana Charles McCoy, Evan D. Peet, Majid Ezzati, Goodarz Danaei, Maureen M. Black, Christopher R. Sudfeld, Wafaie Fawzi, Günther Fink

The following corrections referenced within the text correspond to the PDF version of this article.

On page 2, paragraph 2 within the Abstract under the heading Methods and Findings the authors have revised the following sentence:

Applying this model to all LMICs, we estimate that 81.0 million children ages 3 and 4 y (95% CI 49.2 million, 113.3 million) in LMICs experienced low cognitive and/or socioemotional development in 2010, with the largest number of affected children in sub-Saharan Africa (29.5 million; 44.0% of children ages 3 and 4 y), followed by South Asia (27.8 million; 37.8%) and the East Asia and Pacific region (15.2 million; 26.0%).

Beginning on page 13, paragraph 5 of the Results, the authors have revised the following sentences:

In Table 4, we show our global estimates of the number and percentage of children with low cognitive and/or socioemotional development, which suggests that 81.0 million 3- and 4-y-old children (95% CI 49.2 million, 113.3 million) experienced low cognitive and/or socioemotional development in 2010 as measured by the ECDI. This corresponds to a global prevalence in LMICs of 33.0% (95% CI 20.0%, 46.2%). The highest prevalences of low cognitive and/or socioemotional development were estimated for sub-Saharan Africa (44.0%; 95% CI 30.8%, 57.1%) and South Asia (37.8%; 95% CI 24.7%, 51.0%), whereas the lowest prevalences were estimated for the Latin America/Caribbean region (18.7%; 95% CI 6.1%, 31.9%) and the North Africa/Middle East/Central Asia region (18.5%, 95% CI 6.5%, 31.6%). Sub-Saharan Africa and South Asia also account for the majority of predicted children with low development, with an estimated 29.5 (95% CI 20.7, 38.3) and 27.8 (95% CI 18.1, 37.4) million 3- and 4-y-olds, respectively.

On page 14, paragraph 6 of the Results, the authors have revised the following sentences:

The country with the highest estimated number of children with low development was India (17.2 million children), followed by China (6.7 million) and Nigeria (6.0 million). The estimated percentage of children with low cognitive and/or socioemotional development ranged from as few as 4.8% of children in Qatar to 67% of children in Chad.

On page 15, paragraph 2 of the Discussion, the authors have revised 49.6% to 49.7%.

Errors within the reporting of these datasets have affected Tables 3 and 4 respectively. The authors have provided the corrected tables below.

**Table 3:** Regression models predicting country-level prevalence of low ECDI scores.

	Percentage of children with low cognitive and/or socioemotional ECDI scores		
	Model 1	Model 2	Model 3
Stunting proportion 2010	0.817***		0.0785
	(0.118)		(0.187)
Human Development Index 2010		-1.066***	-0.990***
		(0.112)	(0.195)
Observations	35	35	35
R-squared	0.545	0.711	0.712
Cross validation with n-1 (RMSE)^a^	0.12	0.09	0.10
Cross validation with n-2 (RMSE)^b^	0.08	0.07	0.07

**Notes**:

*** p < .001.

Robust standard errors in parentheses. Column 1 shows a linear model that predicts the proportion of children scoring low on the ECDI based on the NIMS stunting data only. Column 2 shows a model using HDI as the only predictor. Column 3 shows a model including both predictors. All estimates reflect OLS estimates with robust standard errors.

^a^ Based on all 35 possible permutations of size 34.

^b^ Based on 595 permutations of sample size 30.

**Table 4:** Estimated number of 3- and 4-year-olds with low ECDI scores by region.

	Total population ages 3 and 4 in millions	Percentage of children with low cognitive and/or socioemotional ECDI scores (95% CIs)		Number of children with low cognitive and/or socioemotional ECDI scores in millions (95% CIs)	
East Asia/Pacific	58.5	26.0	(12.8, 39.1)	15.2	(7.5, 22.9)
Latin America/Caribbean	21.9	18.7	(6.1, 31.9)	4.1	(1.3, 7)
Africa/Middle East/Central Asia	24.5	18.5	(6.5, 31.6)	4.5	(1.6, 7.8)
South Asia	73.4	37.8	(24.7, 51.0)	27.8	(18.1, 37.4)
Sub-Saharan Africa	67.0	44.0	(30.8, 57.1)	29.5	(20.7, 38.3)
*Total LMICs*	*245*.*3*	33.0	(20.0, 46.2)	*81*.*0*	(49.2, 113.3)

**Notes**: Confidence intervals based on root mean squared errors computed in Table 3. Population numbers are based on the number of children born by country and year in 2010 as reported in the World Population Prospects 2015 edition.

Fig 7 is incorrect. The authors have provided a corrected version here.

**Fig 7:**
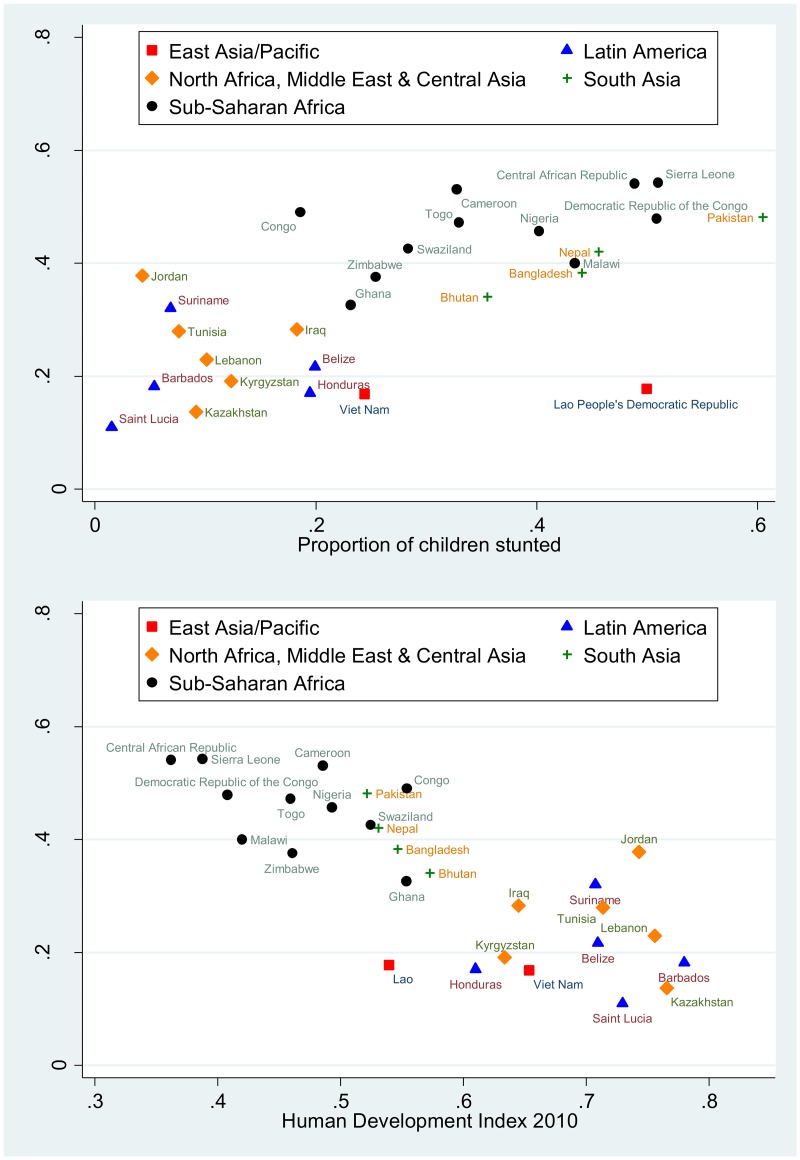
Scatterplots showing country-level relationships between low socioemotional and/or cognitive ECDI score and stunting and HDI. Proportion of children with low socioemotional and/or cognitive ECDI score relative to the proportion of children with stunting (top) and relative to country HDI (bottom).

S3 and S4 Tables contained incorrect datasets. The authors have provided the corrected tables below.

## Supporting Information

S3 Table MICS/DHS versus non-MICS/DHS low- and middle-income country characteristics. This file includes corrected datasets for S3 Table.
(DOCX)

S4 Table Estimated percentage and number of children with low ECDI scores by country. This file includes corrected datasets for S4 Table.
(DOCX)
