# Supplementary figures and images for: Early Childhood Developmental Status in Low- and Middle-Income Countries: National, Regional, and Global Prevalence Estimates Using Predictive Modeling
Source: PLoS Med. 2016 Jun 7;13(6):e1002034. doi: 10.1371/journal.pmed.1002034 (PMC4896459; doi:10.1371/journal.pmed.1002034)

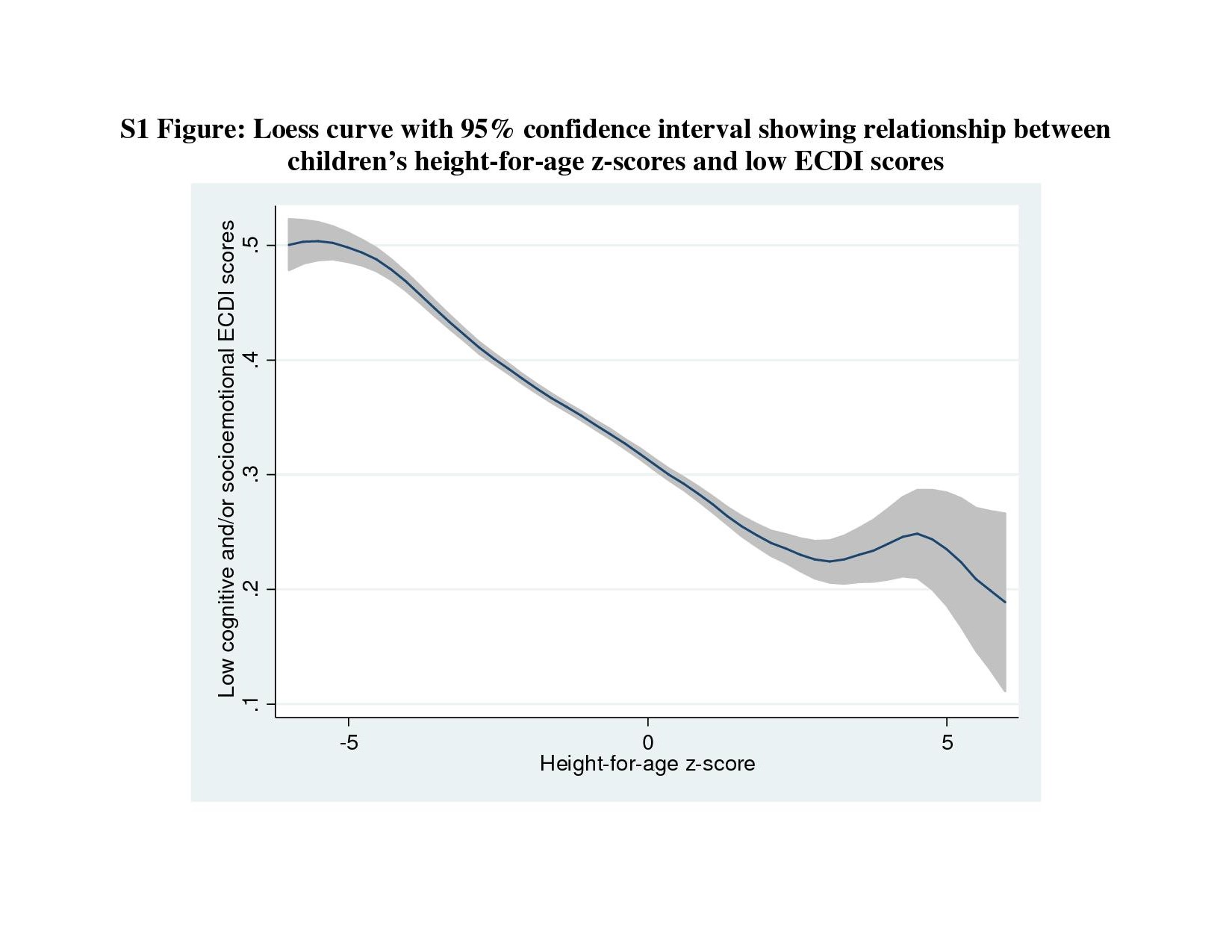

Supplement: S1 Fig — (JPG) [file pmed.1002034.s001.jpg]
