# Supplementary material for: Early Childhood Developmental Status in Low- and Middle-Income Countries: National, Regional, and Global Prevalence Estimates Using Predictive Modeling
Source: PLoS Med. 2016 Jun 7;13(6):e1002034. doi: 10.1371/journal.pmed.1002034 (PMC4896459; doi:10.1371/journal.pmed.1002034)
Supplement: S1 Table — (DOCX) [file pmed.1002034.s002.docx]

**S1 Table: Sample composition**

|  | Total N | Females | | Urban | | Stunted | |
| --- | --- | --- | --- | --- | --- | --- | --- |
|  |  | N | (%) | N | (%) | N | (%) |
| Bangladesh | 7,713 | 3709 | 48.1% | 1237 | 16.0% | 3383 | 43.9% |
| Barbados | 171 | 80 | 46.8% | 97 | 56.7% | 10 | 5.8% |
| Belize | 719 | 361 | 50.2% | 241 | 33.5% | 149 | 20.7% |
| Bhutan | 2,200 | 1074 | 48.8% | 503 | 22.9% | 802 | 36.5% |
| Bosnia | 963 | 498 | 51.7% | 328 | 34.1% | 42 | 4.4% |
| Cameroon^a^ | 1,587 | 809 | 51.0% | 688 | 43.4% | 511 | 32.2% |
| Central African Republic | 3,358 | 1744 | 51.9% | 1126 | 33.5% | 1585 | 47.2% |
| Chad | 4,451 | 2272 | 51.0% | 1758 | 39.5% | 1835 | 41.2% |
| Congo, Democratic Republic | 3,726 | 1870 | 50.2% | 1442 | 38.7% | 1882 | 50.5% |
| Congo^a^ | 1,486 | 735 | 49.5% | 341 | 22.9% | 356 | 24.0% |
| Ghana | 2,928 | 1450 | 49.5% | 843 | 28.8% | 825 | 28.2% |
| Honduras^a^ | 2,800 | 1326 | 47.4% | 977 | 34.9% | 635 | 22.7% |
| Iraq | 13,119 | 6459 | 49.2% | 6981 | 53.2% | 2346 | 17.9% |
| Jordan ^a^ | 2,597 | 1278 | 49.2% | 1849 | 71.2% | 145 | 5.6% |
| Kazakhstan | 1,686 | 802 | 47.6% | 846 | 50.2% | 161 | 9.5% |
| Kosovo | 595 | 286 | 48.1% | 211 | 35.5% | 30 | 5.0% |
| Kyrgyzstan | 1,683 | 822 | 48.8% | 495 | 29.4% | 226 | 13.4% |
| Laos | 4,052 | 1977 | 48.8% | 733 | 18.1% | 2158 | 53.3% |
| Lebanon | 695 | 325 | 46.8% | 442 | 63.6% | 70 | 10.1% |
| Macedonia | 523 | 261 | 49.9% | 285 | 54.5% | 31 | 5.9% |
| Malawi | 7,330 | 3678 | 50.2% | 816 | 11.1% | 3094 | 42.2% |
| Moldova | 620 | 289 | 46.6% | 336 | 54.2% | 39 | 6.3% |
| Montenegro | 1,206 | 554 | 45.9% | 762 | 63.2% | 58 | 4.8% |
| Nepal | 2,142 | 1030 | 48.1% | 377 | 17.6% | 1041 | 48.6% |
| Nigeria | 9,382 | 4576 | 48.8% | 1869 | 19.9% | 4141 | 44.1% |
| Pakistan | 1,463 | 630 | 43.1% | 309 | 21.1% | 861 | 58.9% |
| Serbia | 3,193 | 1570 | 49.2% | 1936 | 60.6% | 101 | 3.2% |
| Sierra Leone | 3,232 | 1604 | 49.6% | 905 | 28.0% | 1655 | 51.2% |
| St. Lucia | 113 | 58 | 51.3% | 40 | 35.4% | 2 | 1.8% |
| Suriname | 997 | 514 | 51.6% | 312 | 31.3% | 82 | 8.2% |
| Swaziland | 1,011 | 523 | 51.7% | 240 | 23.7% | 285 | 28.2% |
| Togo | 1,669 | 814 | 48.8% | 400 | 24.0% | 612 | 36.7% |
| Tunisia | 1,024 | 467 | 45.6% | 598 | 58.4% | 84 | 8.2% |
| Vietnam | 1,366 | 681 | 49.9% | 525 | 38.4% | 340 | 24.9% |
| Zimbabwe | 7,422 | 3732 | 50.3% | 1964 | 26.5% | 1882 | 25.4% |
| *Total* | *99,222* | *48,858* | *49.0%* | *32,812* | *37.0%* | *31,459* | *24.9%* |

**Notes**: ^a^ Data taken from the DHS. All other data were taken from the MICS. All numbers represent unweighted sample averages.
