# Supplementary material for: Early Childhood Developmental Status in Low- and Middle-Income Countries: National, Regional, and Global Prevalence Estimates Using Predictive Modeling
Source: PLoS Med. 2016 Jun 7;13(6):e1002034. doi: 10.1371/journal.pmed.1002034 (PMC4896459; doi:10.1371/journal.pmed.1002034)
Supplement: S2 Table — (DOCX) [file pmed.1002034.s003.docx]

**S2 Table: Factor loadings for ECDI in the full analytic sample**

|  |  | Factor Loading^a^ | *p*-value |
| --- | --- | --- | --- |
| *Literacy & Numeracy* | |  |  |
|  | Letter identification | .75 | <.001 |
|  | Reading | .78 | <.001 |
|  | Number identification | .72 | <.001 |
| *Learning* | |  |  |
|  | Follow simple directions | .78 | <.001 |
|  | Do something independently | .74 | <.001 |
| *Physical* | |  |  |
|  | Too sick to play (*reverse coded*) | .59 | <.001 |
|  | Pick up object with two fingers | .60 | <.001 |
| *Socioemotional* | |  |  |
|  | Kick, bite, or hit (*reverse coded*) | .62 | <.001 |
|  | Easily distracted (*reverse coded*) | .48 | <.001 |
|  | Get along with others | .71 | <.001 |

**Notes**: ^a^ Analyses conducted using confirmatory factor analysis. Factor loadings are standardized, and therefore represent the correlation of each item with the underlying (latent) factor.
