# Supplementary material for: Early Childhood Developmental Status in Low- and Middle-Income Countries: National, Regional, and Global Prevalence Estimates Using Predictive Modeling
Source: PLoS Med. 2016 Jun 7;13(6):e1002034. doi: 10.1371/journal.pmed.1002034 (PMC4896459; doi:10.1371/journal.pmed.1002034)
Supplement: S3 Table — (DOCX) [file pmed.1002034.s004.docx]

**S3 Table: MICS/DHS vs. non-MICS/DHS LMIC characteristics**

|  | MICS/DHS Sample (N=34) | | Non-MICS/DHS LMIC Sample (N=103) | | Two sample equal means test |
| --- | --- | --- | --- | --- | --- |
|  | Mean | (SD) | Mean | (SD) | *p*-value |
| Percentage stunted | 24.61 | 17.69 | 24.42 | 13.20 | 0.96 |
| Human development index 2014 | 0.61 | 0.13 | 0.64 | 0.13 | 0.33 |
| Life expectancy 2014 | 66.38 | 8.77 | 68.88 | 7.11 | 0.13 |
| Average years of schooling 2014 | 7.04 | 2.69 | 6.91 | 2.70 | 0.81 |
| Gross national income 2014 (ln) | 8.51 | 0.98 | 8.77 | 1.09 | 0.19 |

**Notes**: All data are from the 2015 Human Development Index report. No data was available for Kosovo. P-values are based on two-sample means test with robust standard errors.
