# Supplementary material for: Early Childhood Developmental Status in Low- and Middle-Income Countries: National, Regional, and Global Prevalence Estimates Using Predictive Modeling
Source: PLoS Med. 2016 Jun 7;13(6):e1002034. doi: 10.1371/journal.pmed.1002034 (PMC4896459; doi:10.1371/journal.pmed.1002034)
Supplement: S4 Table — (DOCX) [file pmed.1002034.s005.docx]

**S4 Table: Estimated percentage and number of children with low ECDI scores by country**

| Country | Estimated percentage of 3- and 4-year-old children with low ECDI scores | Type of estimate | Number of 3- and 4-year-old children (in ,000s) with low ECDI scores |
| --- | --- | --- | --- |
| Afghanistan | 46.9% | Model prediction | 1,024.8 |
| Algeria | 17.4% | Model prediction | 304.1 |
| Angola | 40.4% | Model prediction | 817.3 |
| Antigua and Barbuda | 11.3% | Model prediction | 0.3 |
| Argentina | 8.3% | Model prediction | 124.1 |
| Armenia | 17.8% | Model prediction | 14.7 |
| Azerbaijan | 15.8% | Model prediction | 60.8 |
| Bahamas | 12.2% | Model prediction | 1.4 |
| Bahrain | 7.4% | Model prediction | 2.8 |
| Bangladesh | 38.3% | MICS/DHS | 2,490.2 |
| Barbados | 18.2% | MICS/DHS | 1.3 |
| Belize | 21.6% | MICS/DHS | 3.3 |
| Benin | 44.8% | Model prediction | 322.4 |
| Bhutan | 34.1% | MICS/DHS | 9.8 |
| Bolivia | 26.3% | Model prediction | 133.5 |
| Botswana | 4.4% | MICS/DHS | 4.6 |
| Brazil | 16.1% | Model prediction | 1,006.2 |
| Burkina Faso | 54.3% | Model prediction | 718.5 |
| Burundi | 53.1% | Model prediction | 446.5 |
| Cambodia | 37.5% | Model prediction | 274.2 |
| Cameroon | 53.1% | MICS/DHS | 844.6 |
| Cape Verde | 27.6% | Model prediction | 6.1 |
| Central African Republic | 54.1% | MICS/DHS | 168.6 |
| Chad | 67.0% | MICS/DHS | 755.5 |
| Chile | 8.0% | Model prediction | 38.0 |
| China | 20.2% | Model prediction | 6,667.9 |
| Colombia | 19.5% | Model prediction | 305.1 |
| Comoros | 42.6% | Model prediction | 21.0 |
| Congo | 49.0% | MICS/DHS | 151.0 |
| Costa Rica | 14.8% | Model prediction | 21.2 |
| Cuba | 11.8% | Model prediction | 29.1 |
| Cote d'Ivoire | 47.2% | Model prediction | 725.8 |
| Democratic Republic of the Congo | 47.9% | MICS/DHS | 2,770.3 |
| Djibouti | 46.4% | Model prediction | 20.5 |
| Dominican Republic | 20.0% | Model prediction | 87.6 |
| Ecuador | 18.3% | Model prediction | 119.5 |
| Egypt | 22.1% | Model prediction | 985.1 |
| El Salvador | 25.1% | Model prediction | 55.7 |
| Equatorial Guinea | 31.7% | Model prediction | 16.8 |
| Eritrea | 54.0% | Model prediction | 184.5 |
| Ethiopia | 50.7% | Model prediction | 3,091.2 |
| Fiji | 18.3% | Model prediction | 6.8 |
| Gabon | 24.0% | Model prediction | 23.1 |
| Gambia | 47.6% | Model prediction | 70.1 |
| Georgia | 16.4% | Model prediction | 19.2 |
| Ghana | 32.6% | MICS/DHS | 532.1 |
| Grenada | 16.1% | Model prediction | 0.7 |
| Guatemala | 29.5% | Model prediction | 249.6 |
| Guinea | 53.3% | Model prediction | 455.6 |
| Guinea-Bissau | 50.6% | Model prediction | 63.8 |
| Guyana | 28.2% | Model prediction | 8.2 |
| Haiti | 44.5% | Model prediction | 236.7 |
| Honduras | 17.0% | MICS/DHS | 59.7 |
| India | 32.2% | Model prediction | 17,147.5 |
| Indonesia | 23.8% | Model prediction | 2,409.0 |
| Iran (Islamic Republic of) | 15.5% | Model prediction | 417.6 |
| Iraq | 28.3% | MICS/DHS | 625.2 |
| Jamaica | 17.2% | Model prediction | 17.1 |
| Jordan | 37.8% | MICS/DHS | 138.8 |
| Kazakhstan | 13.6% | MICS/DHS | 99.1 |
| Kenya | 38.3% | Model prediction | 1,134.5 |
| Kiribati | 32.0% | Model prediction | 1.9 |
| Kuwait | 8.5% | Model prediction | 11.4 |
| Kyrgyzstan | 19.1% | MICS/DHS | 53.7 |
| Lao People's Democratic Republic | 17.7% | MICS/DHS | 62.4 |
| Lebanon | 22.9% | MICS/DHS | 29.6 |
| Lesotho | 44.4% | Model prediction | 51.4 |
| Liberia | 51.5% | Model prediction | 149.7 |
| Libyan Arab Jamahiriya | 14.1% | Model prediction | 38.7 |
| Madagascar | 40.9% | Model prediction | 615.1 |
| Malawi | 40.0% | MICS/DHS | 486.7 |
| Malaysia | 12.7% | Model prediction | 121.0 |
| Maldives | 21.9% | Model prediction | 3.1 |
| Mali | 51.0% | Model prediction | 707.9 |
| Mauritania | 42.7% | Model prediction | 107.3 |
| Mauritius | 14.2% | Model prediction | 4.3 |
| Mexico | 15.2% | Model prediction | 723.8 |
| Micronesia (Federated States of) | 26.7% | Model prediction | 1.3 |
| Mongolia | 20.6% | Model prediction | 26.3 |
| Morocco | 29.6% | Model prediction | 401.0 |
| Mozambique | 51.9% | Model prediction | 1,037.3 |
| Myanmar | 39.2% | Model prediction | 799.8 |
| Namibia | 29.6% | Model prediction | 39.2 |
| Nepal | 42.0% | MICS/DHS | 522.8 |
| Nicaragua | 28.7% | Model prediction | 72.9 |
| Niger | 59.9% | Model prediction | 992.5 |
| Nigeria | 45.7% | MICS/DHS | 5,999.5 |
| Occupied Palestinian Territory | 23.3% | Model prediction | 64.0 |
| Oman | 10.0% | Model prediction | 13.6 |
| Pakistan | 48.1% | MICS/DHS | 4,928.8 |
| Panama | 13.6% | Model prediction | 20.1 |
| Papua New Guinea | 42.1% | Model prediction | 174.0 |
| Paraguay | 23.5% | Model prediction | 65.5 |
| Peru | 18.2% | Model prediction | 223.6 |
| Philippines | 24.9% | Model prediction | 1,150.5 |
| Qatar | 4.8% | Model prediction | 2.0 |
| Rwanda | 46.3% | Model prediction | 335.1 |
| Saint Lucia | 11.0% | MICS/DHS | 0.6 |
| Saint Vincent and the Grenadines | 18.9% | Model prediction | 0.7 |
| Samoa | 20.5% | Model prediction | 2.1 |
| Sao Tome and Principe | 36.7% | Model prediction | 4.5 |
| Saudi Arabia | 9.0% | Model prediction | 109.1 |
| Senegal | 46.0% | Model prediction | 468.7 |
| Seychelles | 15.5% | Model prediction | 0.5 |
| Sierra Leone | 54.3% | MICS/DHS | 244.3 |
| Solomon Islands | 42.0% | Model prediction | 14.3 |
| South Africa | 26.1% | Model prediction | 579.9 |
| Sri Lanka | 16.0% | Model prediction | 112.3 |
| Sudan | 45.0% | Model prediction | 1,133.3 |
| Suriname | 32.0% | MICS/DHS | 6.4 |
| Swaziland | 42.5% | MICS/DHS | 31.3 |
| Syrian Arab Republic | 26.6% | Model prediction | 261.4 |
| Tajikistan | 29.9% | Model prediction | 137.9 |
| Thailand | 18.4% | Model prediction | 288.0 |
| Timor-Leste | 30.7% | Model prediction | 26.0 |
| Togo | 47.3% | MICS/DHS | 226.9 |
| Tonga | 18.7% | Model prediction | 1.0 |
| Trinidad and Tobago | 12.5% | Model prediction | 5.0 |
| Tunisia | 27.9% | MICS/DHS | 105.5 |
| Turkey | 16.1% | Model prediction | 420.1 |
| Turkmenistan | 23.7% | Model prediction | 52.1 |
| Uganda | 44.2% | Model prediction | 1,321.1 |
| United Arab Emirates | 6.4% | Model prediction | 11.5 |
| United Republic of Tanzania | 41.4% | Model prediction | 1,549.4 |
| Uruguay | 11.6% | Model prediction | 11.5 |
| Uzbekistan | 24.9% | Model prediction | 317.9 |
| Vanuatu | 31.8% | Model prediction | 4.2 |
| Venezuela (Bolivarian Republic of) | 14.0% | Model prediction | 168.1 |
| Viet Nam | 16.8% | MICS/DHS | 516.8 |
| Yemen | 41.7% | Model prediction | 678.6 |
| Zambia | 35.5% | Model prediction | 416.1 |
| Zimbabwe | 37.5% | MICS/DHS | 380.2 |

**Notes**: Population numbers based on World Population Prospects 2015. Estimated prevalence of low ECDI scores is based on MICS estimates where data are available and based on predictive model 2 otherwise. Countries from Eastern Europe were not included in the global LMIC model due to the lack of anthropometric data.
