# Supplementary material for: Correction: Early Childhood Developmental Status in Low- and Middle-Income Countries: National, Regional, and Global Prevalence Estimates Using Predictive Modelling
Source: PLoS Med. 2017 Jan 30;14(1):e1002233. doi: 10.1371/journal.pmed.1002233 (PMC5279732; doi:10.1371/journal.pmed.1002233)
Supplement: S4 Table — This file includes corrected datasets for S4 Table. (DOCX) [file pmed.1002233.s002.docx]

**S4 Table: Estimated percentage and number of children with low ECDI scores by country**

| Country | Estimated percentage of children with low ECDI scores | Type of estimate | Number of children age 3 and 4 with low ECDI scores |
| --- | --- | --- | --- |
| Afghanistan | 47.0% | Model prediction | 1,027.7 |
| Algeria | 17.4% | Model prediction | 305.0 |
| Angola | 40.5% | Model prediction | 819.6 |
| Antigua and Barbuda | 11.4% | Model prediction | 0.3 |
| Argentina | 8.3% | Model prediction | 124.4 |
| Armenia | 17.9% | Model prediction | 14.7 |
| Azerbaijan | 15.8% | Model prediction | 61.0 |
| Bahamas | 12.3% | Model prediction | 1.4 |
| Bahrain | 7.4% | Model prediction | 2.8 |
| Bangladesh | 38.3% | MICS/DHS | 2,490.2 |
| Barbados | 18.2% | MICS/DHS | 1.3 |
| Belize | 21.6% | MICS/DHS | 3.3 |
| Benin | 44.9% | Model prediction | 323.3 |
| Bhutan | 34.1% | MICS/DHS | 9.8 |
| Bolivia | 26.4% | Model prediction | 133.8 |
| Botswana | 22.2% | Model prediction | 23.2 |
| Brazil | 16.2% | Model prediction | 1,009.2 |
| Burkina Faso | 54.5% | Model prediction | 720.5 |
| Burundi | 53.2% | Model prediction | 447.8 |
| Cambodia | 37.6% | Model prediction | 275.0 |
| Cameroon | 53.1% | MICS/DHS | 844.6 |
| Cape Verde | 27.7% | Model prediction | 6.1 |
| Central African Republic | 54.1% | MICS/DHS | 168.6 |
| Chad | 67.0% | MICS/DHS | 755.5 |
| Chile | 8.0% | Model prediction | 38.1 |
| China | 20.3% | Model prediction | 6,687.4 |
| Colombia | 19.5% | Model prediction | 306.0 |
| Comoros | 42.7% | Model prediction | 21.1 |
| Congo | 49.0% | MICS/DHS | 151.0 |
| Costa Rica | 14.8% | Model prediction | 21.3 |
| Cuba | 11.9% | Model prediction | 29.2 |
| Cote d'Ivoire | 47.4% | Model prediction | 727.8 |
| Democratic Republic of the Congo | 47.9% | MICS/DHS | 2,770.3 |
| Djibouti | 46.5% | Model prediction | 20.6 |
| Dominican Republic | 20.0% | Model prediction | 87.9 |
| Ecuador | 18.4% | Model prediction | 119.8 |
| Egypt | 22.2% | Model prediction | 988.0 |
| El Salvador | 25.2% | Model prediction | 55.9 |
| Equatorial Guinea | 31.8% | Model prediction | 16.8 |
| Eritrea | 54.1% | Model prediction | 185.1 |
| Ethiopia | 50.9% | Model prediction | 3,100.0 |
| Fiji | 18.3% | Model prediction | 6.8 |
| Gabon | 24.1% | Model prediction | 23.2 |
| Gambia | 47.8% | Model prediction | 70.3 |
| Georgia | 16.4% | Model prediction | 19.2 |
| Ghana | 32.6% | MICS/DHS | 532.1 |
| Grenada | 16.2% | Model prediction | 0.7 |
| Guatemala | 29.6% | Model prediction | 250.3 |
| Guinea | 53.5% | Model prediction | 456.9 |
| Guinea-Bissau | 50.8% | Model prediction | 64.0 |
| Guyana | 28.3% | Model prediction | 8.2 |
| Haiti | 44.6% | Model prediction | 237.3 |
| Honduras | 17.0% | MICS/DHS | 59.7 |
| India | 32.3% | Model prediction | 17,196.9 |
| Indonesia | 23.9% | Model prediction | 2,416.0 |
| Iran (Islamic Republic of) | 15.5% | Model prediction | 418.9 |
| Iraq | 28.3% | MICS/DHS | 625.2 |
| Jamaica | 17.3% | Model prediction | 17.1 |
| Jordan | 37.8% | MICS/DHS | 138.8 |
| Kazakhstan | 13.6% | MICS/DHS | 99.1 |
| Kenya | 38.4% | Model prediction | 1,137.7 |
| Kiribati | 32.1% | Model prediction | 1.9 |
| Kuwait | 8.6% | Model prediction | 11.4 |
| Kyrgyzstan | 19.1% | MICS/DHS | 53.7 |
| Lao People's Democratic Republic | 17.7% | MICS/DHS | 62.4 |
| Lebanon | 22.9% | MICS/DHS | 29.6 |
| Lesotho | 44.5% | Model prediction | 51.5 |
| Liberia | 51.6% | Model prediction | 150.1 |
| Libyan Arab Jamahiriya | 14.1% | Model prediction | 38.8 |
| Madagascar | 41.0% | Model prediction | 616.8 |
| Malawi | 40.0% | MICS/DHS | 486.7 |
| Malaysia | 12.7% | Model prediction | 121.3 |
| Maldives | 22.0% | Model prediction | 3.1 |
| Mali | 51.1% | Model prediction | 709.9 |
| Mauritania | 42.8% | Model prediction | 107.6 |
| Mauritius | 14.2% | Model prediction | 4.4 |
| Mexico | 15.3% | Model prediction | 726.0 |
| Micronesia (Federated States of) | 26.7% | Model prediction | 1.3 |
| Mongolia | 20.6% | Model prediction | 26.4 |
| Morocco | 29.6% | Model prediction | 402.2 |
| Mozambique | 52.0% | Model prediction | 1,040.2 |
| Myanmar | 39.3% | Model prediction | 802.0 |
| Namibia | 29.7% | Model prediction | 39.3 |
| Nepal | 42.0% | MICS/DHS | 522.8 |
| Nicaragua | 28.8% | Model prediction | 73.1 |
| Niger | 60.0% | Model prediction | 995.3 |
| Nigeria | 45.7% | MICS/DHS | 5,999.5 |
| Occupied Palestinian Territory | 23.4% | Model prediction | 64.2 |
| Oman | 10.0% | Model prediction | 13.6 |
| Pakistan | 48.1% | MICS/DHS | 4,928.8 |
| Panama | 13.6% | Model prediction | 20.1 |
| Papua New Guinea | 42.2% | Model prediction | 174.5 |
| Paraguay | 23.5% | Model prediction | 65.7 |
| Peru | 18.3% | Model prediction | 224.3 |
| Philippines | 25.0% | Model prediction | 1,153.8 |
| Qatar | 4.8% | Model prediction | 2.0 |
| Rwanda | 46.4% | Model prediction | 336.0 |
| Saint Lucia | 11.0% | MICS/DHS | 0.6 |
| Saint Vincent and the Grenadines | 18.9% | Model prediction | 0.7 |
| Samoa | 20.6% | Model prediction | 2.1 |
| Sao Tome and Principe | 36.8% | Model prediction | 4.5 |
| Saudi Arabia | 9.0% | Model prediction | 109.5 |
| Senegal | 46.2% | Model prediction | 470.0 |
| Seychelles | 15.5% | Model prediction | 0.5 |
| Sierra Leone | 54.3% | MICS/DHS | 244.3 |
| Solomon Islands | 42.1% | Model prediction | 14.3 |
| South Africa | 26.2% | Model prediction | 581.6 |
| Sri Lanka | 16.1% | Model prediction | 112.7 |
| Sudan | 45.2% | Model prediction | 1,136.5 |
| Suriname | 32.0% | MICS/DHS | 6.4 |
| Swaziland | 42.5% | MICS/DHS | 31.3 |
| Syrian Arab Republic | 26.6% | Model prediction | 262.1 |
| Tajikistan | 30.0% | Model prediction | 138.3 |
| Thailand | 18.5% | Model prediction | 288.9 |
| Timor-Leste | 30.8% | Model prediction | 26.0 |
| Togo | 47.3% | MICS/DHS | 226.9 |
| Tonga | 18.8% | Model prediction | 1.0 |
| Trinidad and Tobago | 12.5% | Model prediction | 5.0 |
| Tunisia | 27.9% | MICS/DHS | 105.5 |
| Turkey | 16.1% | Model prediction | 421.3 |
| Turkmenistan | 23.8% | Model prediction | 52.3 |
| Uganda | 44.4% | Model prediction | 1,324.9 |
| United Arab Emirates | 6.5% | Model prediction | 11.5 |
| United Republic of Tanzania | 41.5% | Model prediction | 1,553.9 |
| Uruguay | 11.6% | Model prediction | 11.6 |
| Uzbekistan | 24.9% | Model prediction | 318.8 |
| Vanuatu | 31.9% | Model prediction | 4.2 |
| Venezuela (Bolivarian Republic of) | 14.1% | Model prediction | 168.6 |
| Viet Nam | 16.8% | MICS/DHS | 516.8 |
| Yemen | 41.8% | Model prediction | 680.6 |
| Zambia | 35.6% | Model prediction | 417.3 |
| Zimbabwe | 37.5% | MICS/DHS | 380.2 |

**Notes**: Population numbers based on World Population Prospects 2015. Estimated prevalence of low ECDI scores is based on MICS estimates where data is available and based on predictive model 2 otherwise. Countries from Eastern Europe were not included in the global LMIC model to the lack of anthropometric data.
